# Inter- and Intra-Scanner Variability of Automated Brain Volumetry on Three Magnetic Resonance Imaging Systems in Alzheimer’s Disease and Controls

**DOI:** 10.3389/fnagi.2021.746982

**Published:** 2021-10-07

**Authors:** Mandy Melissa Jane Wittens, Gert-Jan Allemeersch, Diana Maria Sima, Maarten Naeyaert, Tim Vanderhasselt, Anne-Marie Vanbinst, Nico Buls, Yannick De Brucker, Hubert Raeymaekers, Erik Fransen, Dirk Smeets, Wim van Hecke, Guy Nagels, Maria Bjerke, Johan de Mey, Sebastiaan Engelborghs

**Affiliations:** ^1^Reference Center for Biological Markers of Dementia, Laboratory of Neurochemistry and Behavior, University of Antwerp, Antwerp, Belgium; ^2^Center for Neurosciences (C4N) and Department of Neurology, Vrije Universiteit Brussel, Universitair Ziekenhuis Brussel, Brussels, Belgium; ^3^Department of Radiology, Vrije Universiteit Brussel, Universitair Ziekenhuis Brussel, Brussels, Belgium; ^4^Icometrix, Leuven, Belgium; ^5^StatUa Center for Statistics, University of Antwerp, Antwerp, Belgium

**Keywords:** Alzheimer’s disease, m**agnetic** r**esonanc**e i**maging**, automated volumetry, i**nter- and intra-scanner variability**, biomarkers

## Abstract

Magnetic Resonance Imaging (MRI) has become part of the clinical routine for diagnosing neurodegenerative disorders. Since acquisitions are performed at multiple centers using multiple imaging systems, detailed analysis of brain volumetry differences between MRI systems and scan-rescan acquisitions can provide valuable information to correct for different MRI scanner effects in multi-center longitudinal studies. To this end, five healthy controls and five patients belonging to various stages of the AD continuum underwent brain MRI acquisitions on three different MRI systems (Philips Achieva dStream 1.5T, Philips Ingenia 3T, and GE Discovery MR750w 3T) with harmonized scan parameters. Each participant underwent two subsequent MRI scans per imaging system, repeated on three different MRI systems within 2 h. Brain volumes computed by icobrain dm (v5.0) were analyzed using absolute and percentual volume differences, Dice similarity (DSC) and intraclass correlation coefficients, and coefficients of variation (CV). Harmonized scans obtained with different scanners of the same manufacturer had a measurement error closer to the intra-scanner performance. The gap between intra- and inter-scanner comparisons grew when comparing scans from different manufacturers. This was observed at image level (image contrast, similarity, and geometry) and translated into a higher variability of automated brain volumetry. Mixed effects modeling revealed a significant effect of scanner type on some brain volumes, and of the scanner combination on DSC. The study concluded a good intra- and inter-scanner reproducibility, as illustrated by an average intra-scanner (inter-scanner) CV below 2% (5%) and an excellent overlap of brain structure segmentation (mean DSC > 0.88).

## Introduction

The elderly population is drastically increasing and so does the prevalence of dementia. In the last decade, magnetic resonance imaging (MRI) has become an important tool in the diagnostic work-up for patients with Alzheimer’s disease (AD), where MRI scans are essential for detecting brain atrophy and atrophy patterns for differential diagnosis of dementia subtypes (Frisoni et al., 2010). In this context, the introduction of visual rating scales to assess hyperintensities, global cortical atrophy, posterior cortical atrophy, and medial temporal lobe atrophy on brain MRI scans has helped standardizing radiological reading in AD and the differential diagnosis in dementia (Albert et al., 2011; Jack et al., 2011; Dubois et al., 2014; Niemantsverdriet et al., 2018; Struyfs et al., 2020). However, these rating scales are based on the visual assessment of 3D structures through 2D slices. As a result, despite the important role of these scales in the clinical setting, it is known that they are time-consuming, subjective, not uniformly adopted, and dependent on the expertise of the radiologist (Vernooij et al., 2019). Therefore, recent developments in the field of imaging artificial intelligence (AI) have enabled the automatic extraction of clinically relevant measures from brain MRI scans (Niemantsverdriet et al., 2018; Struyfs et al., 2020; Wittens et al., 2021). Thereupon, it has been shown that automated volumetry, combined with the expertise of radiologists, can improve the sensitivity and specificity of assessing AD-related atrophy (Pemberton et al., 2021). Since combining modern AI technology with radiological expertise has the potential to detect and monitor abnormalities more accurately, a strict validation of AI tools is necessary to assess the validity in a clinical setting. However, within and between-scanner variability can at least partially neutralize the added diagnostic value of AI-based automated volumetry for the (differential) diagnosis of AD. Several studies testing the repeatability and reproducibility of different automated volumetric tools for white matter hyperintensity (WMH) quantifications, as well as for brain volumetric measurements in other neurological disorders such as multiple sclerosis, have shown the importance of considering variation when comparing scans from multi-center and longitudinal studies. In addition, the consistent appliance of metrics such as the coefficient of variation (CV), absolute volume differences (AVD), as well as intra-class correlation and Dice similarity coefficients (DSC) in studies assessing intra- and inter-scanner variability, facilitate between study comparisons (Gasperini et al., 2001; Huppertz et al., 2010; Biberacher et al., 2016; Shinohara et al., 2017; Guo et al., 2019). We have therefore set up a study to assess the effect of within- and between-scanner variabilities on three different MRI systems with harmonized scan parameters, using automated volumetry computed by the CE-labeled and FDA-cleared post-processing software icobrain dm (Struyfs et al., 2020). To this end, 10 subjects were scanned twice during a time-interval of 2 h, and automatically computed brain volumes were statistically analyzed to assess intra- and inter-scanner agreement. The purpose of this study is to determine the extent of intra and inter-scanner variability with harmonized acquisition parameters and its implication in routine clinical practice.

## Materials and Methods

### Study Population

The study population included five healthy controls and five patients in different stages of the AD continuum, resulting in a total of ten participants (Table 1). Recruitment of all participants was effectuated at the Neurology and Radiology departments of UZ Brussel between April 2020 and August 2020. The exclusion criteria consisted of defibrillators, neurostimulators, pacemakers, and all other standard MRI contraindications, advanced AD (defined as having a Mini-Mental State Examination score < 10/30), and brain tumors or other neurological disorders that could cause cognitive impairment. Patient classification was effectuated in compliance with the National Institute on Aging-Alzheimer’s Association criteria for “MCI due to AD” and “Dementia due to AD” (Albert et al., 2011; Jack et al., 2011; McKhann et al., 2011; Sperling et al., 2011; Dubois et al., 2014). Four MCI patients, and one dementia due to AD patient were included in this study.

**TABLE 1:** Study population demographics.

	Healthy control	AD continuum	Total	*p*-value
Inclusions (*n*)	5	5	10	–
Age in years (mean ± SD)	52.2 ± 17.6	69.0 ± 10.5	60.6 ± 16.3	0.001
Gender (m/f)	3/2	4/1	7/3	0.490
MMSE (*n*) 0–30	NA	26 ± 2	26 ± 2	–

### Study Design

For this prospective study, three different MRI imaging systems were used (section “Image acquisition,” Table 2). All three MRI scanners are located at the radiology department of the VUB university hospital (UZ Brussel), Brussels, Belgium. To test the intra-scanner variability, each participant underwent two MRI scans per imaging system in a randomized manner. In between the two scans on the same MRI system, the participants were repositioned to make sure that a difference in positioning is considered during the evaluation. The duration time of each MRI scan varied between 6 and 10 min per scanner. The time in between scans was 3–5 min.

**TABLE 2:** 3D T1w and FLAIR sequence scan parameters.

	3D T1		
	Achieva	Ingenia	GE
Sequence	TFE	TFE	BRAVO
Coil	16 channels	32 channels	16 channels
Field of view (mm^3^)	230 × 230 × 172.8	230 × 230 × 172.8	256 × 256 × 172.8
Phase oversampling	12%	12%	/
Acq. resolution (mm^3^)	1.2 × 1.2 × 1.2	1.2 × 1.2 × 1.2	1.2 × 1.2 × 1.2
Reco. resolution (mm^3^)	0.6 × 0.6 × 0.6	0.6 × 0.6 × 0.6	1 × 1 × 0.6
Slice orientation	Sagittal	Sagittal	Sagittal
TE (ms)	4.6	2.8	2.8
TR (ms)	8.5	7.6	7.6
TI (ms)	888	800	800
Bandwidth (Hz/pixel)	217.6	309.6	294.8
TFE factor / ETL	144	144	/
Flip angle	8°	8°	8°
Acceleration technique	Compressed sense	Compressed sense	SENSE
Acceleration factor	2	2	2 × 1
Scan time	2 min 34 s	2 min 44 s	3 min 45 s

	**FLAIR**		
	**Achieva**	**Ingenia**	**GE**

Sequence	TSE	TSE	CUBE
Coil	16 channels	32 channels	16 channels
Field of view (mm^3^)	230 × 230 × 172.8	230 × 230 × 172.8	256 × 256 × 172.8
Phase oversampling	12%	12%	/
Acq. resolution (mm^3^)	1.2 × 1.2 × 1.2	1.2 × 1.2 × 1.2	1.2 × 1.2 × 1.2
Reco. resolution (mm^3^)	0.6 × 0.6 × 0.6	0.6 × 0.6 × 0.6	1 × 1 × 0.6
Slice orientation	Sagittal	Sagittal	Sagittal
TE (ms)	154	155	126
TR (ms)	4,800	5,000	5,000
TI (ms)	1,650	1,650	1,466
bandwidth (Hz/pixel)	440	867.1	589.6
TFE factor / ETL	230	230	230
Acceleration technique	Compressed sense	Compressed sense	SENSE
Acceleration factor	4	4	2 × 2
Scan time	4 min 20 s	4 min 35 s	3 min 3 s

*Reconstruction parameters were kept similar where possible, with voxel interpolation not exceeding a factor of 2 in any dimension. **Achieva:** Philips Medical Systems Achieva dStream 1.5T. **Ingenia:** Philips Medical Systems Ingenia 3T. **GE:** GE Discovery MR750w 3T.*

To test the inter-scanner variability, this workflow was repeated on three different MRI systems. A total of sixty MRI scans were used for downstream comparative analysis. The total scan time for all systems combined was a maximum of 90 min per participant. To minimize the variability in brain volume, all scans were performed in a time span of 2 h.

### Ethical Committee

This randomized prospective study was approved by the Ethical Committee of UZ Brussel in Brussels, Belgium (Reference nr: 2020-079). Written informed consent of all participants and/or legal representatives (in case of dementia) was obtained.

### Image Acquisition

All subjects were scanned twice on each of the three following scanners: a 1.5T Achieva dStream (Philips Medical Systems, Best, Netherlands), 3T Ingenia (Philips Medical Systems, Best, Netherlands) and a Discovery MR750w 3T (GE Medical Systems, Milwaukee, WI, United States), and will be further referred to as “Achieva,” “Ingenia,” and “GE.” Every MRI scan study consisted of a sagittal 3D T1-weighted (T1w) MR sequence and a sagittal 3D Fluid Attenuated Inversion Recovery (FLAIR) sequence. Sequence parameters were harmonized as much as possible between the vendors, limited by the constraints of each manufacturer. Priority was given to harmonization of acquisition parameters over reconstruction parameters. The parameters are based on the existing clinical routine scans for AD within UZ Brussel. The 3T Ingenia scanner parameters were taken as a starting point and were subsequently modified on the 1.5T Achieva and Discovery MR750w 3T MRI systems to be as alike as possible. This was done through harmonizing resolution, timings, flip angles, and bandwidth. Test scans were performed on volunteers on the various systems and checked by two radiologists (G-JA and TV). All scans were visually investigated and feedback was provided. If necessary, the parameters were adapted and the procedure was repeated. No quantification was performed during testing since T1 and FLAIR data were already being quantified by Icometrix as part of the clinical routine and positive feedback on the scan quality was obtained. Lastly, voxel interpolation during reconstruction did not exceed a factor 2 in any dimension. All scan parameters are listed in Table 2.

#### Image Processing

A visual assessment was performed to exclude possible causes of inaccurate measurements, including, but not limited to, motion artifacts, metal artifacts, and head-coil artifacts.

#### Post Processing Technique

All sixty MRI-scans were processed with icobrain dm by icometrix, Leuven, Belgium. icobrain dm (version 5.0) is a medical device software providing automated volumetric analysis of global and local brain region volumes. In short, after skull stripping and bias field correction, the icobrain pipeline performs an initial segmentation into gray matter (GM), white matter (WM), and cerebrospinal fluid (CSF; including WMH if a FLAIR is available). This step is further refined to obtain sub-segmentations such as the hippocampi and cortical gray matter (CGM) volumes.

Brain structure volumes relevant for (differential) AD diagnosis, such as the frontal cortex (Harper et al., 2017; Sawyer et al., 2017; Cajanus et al., 2019), parietal cortex (PC; Jacobs et al., 2012b; Harper et al., 2016; Peng et al., 2016), temporal cortex (TC; Duara et al., 2008; Vemuri and Jack, 2010; Clerx et al., 2013; Harper et al., 2016), total hippocampal volume (HIP-T; Den Heijer et al., 2010; Vemuri and Jack, 2010; Jacobs et al., 2012a; Rathakrishnan et al., 2014; Halliday, 2017; Martinez-Torteya et al., 2019), left hippocampus (HIP-L; Lindberg et al., 2012; Maruszak and Thuret, 2014; Sarica et al., 2018; Martinez-Torteya et al., 2019; Struyfs et al., 2020; Wittens et al., 2021), right hippocampus (HIP-R; Maruszak and Thuret, 2014; Sarica et al., 2018; Struyfs et al., 2020; Wittens et al., 2021), and lateral ventricles (LVENT; Guptha et al., 2002; Ferrarini et al., 2006; Apostolova et al., 2012; Struyfs et al., 2020; Wittens et al., 2021), were analyzed for each of the three scanner types. Larger brain structure volumes such as whole brain, GM, CGM and WM were also analyzed to assess differences in measurement error that can be ascribed to differences in brain structure volumes.

For details regarding icobrain dm’s pipeline, including the cortical lobe, and hippocampal segmentation procedure, we refer to Jain et al. (2015), Struyfs et al. (2020), and Wittens et al. (2021).

### Statistical Analysis

All data processing was performed using the R environment (R-Studio, v.1.0.136) for statistical computing and graphics using the following “packages” and (functions). Demographic information was reported as mean and standard deviation (SD; where applicable), with a significance level of <0.05 [R package: “arsenal” (tableby and write2word)].

#### Measures of Agreement at Image Level

The similarity between pairs of T1w scans is reported using an affine similarity index, contrast difference between two T1w images of the same subject, and a maximum scaling factor.

##### Affine similarity index

An affine similarity index is defined as the normalized mutual information (NMI) between any two T1w images of the same subject after affine registration between the two images (Studholme et al., 1999). This measure expresses how well two images match without requiring that the image intensities are similar, thus it is a measure of scan similarity that can be assessed in a clinical setting and can be related to the measurement error. An affine registration allows an image to be mapped to another image using rotation, translation, scaling and skewing. As it is a global transformation, the same rotation, translation, scaling, and skewing parameters are applied for the entire image, meaning that there are no different parameters for different voxels or structures. Post alignment, the NMI is calculated, where higher NMI values express stronger similarity and lower values express more mismatch. Previously, it was found that the alignment between two T1w images can be considered reliable when the affine similarity index is above 0.2 (Sima et al., 2019).

##### Maximum scaling factor

A maximum scaling factor is also reported as the maximal stretching along any of the three spatial axes when affinely registering two T1w images of the same subject. A value above one might indicate that there are geometric differences between the two T1w images, while a maximum scaling factor of 1 indicates that no scaling is needed in any of the three directions to perfectly align the two images. For the sake of simplicity, we do not differentiate between stretching and shrinking because these are inverse operations, depending on which image is considered as reference.

##### Contrast difference

Besides measuring global image similarity between pairs of T1w scans with the affine similarity index, the contrast difference between two T1w images of the same subject is also computed. The image contrast is defined as the contrast-to-noise ratio (CNR) between WM and GM image intensities (Magnotta and Friedman, 2006), computed as:


$$C\,N\,R=\frac{\left|m\,e\,a\,n\,i\,n\,t\,e\,n\,s\,i\,t\,y\,G\,M-m\,e\,a\,n\,i\,n\,t\,e\,n\,s\,i\,t\,y\,W\,M\right|}{\sqrt{v\,a\,r\,i\,a\,n\,c\,e\,i\,n\,t\,e\,n\,s\,i\,t\,y\,G\,M+v\,a\,r\,i\,a\,n\,c\,e\,i\,n\,t\,e\,n\,s\,i\,t\,y\,W\,M}}$$

It is expected that images with similar contrast, as indicated by a lower absolute difference in WM/GM CNR, would be segmented more consistently.

#### Measures of Agreement for Intra- and Inter-Scanner Brain Volumes and Segmentations

##### Intraclass correlation coefficient

The intra-scanner variability was analyzed by determining the intraclass correlation coefficient (ICC, with 95% CI), using the function “ICC” of R package psych (v. 2.3.0), based on absolute agreement, single-measurement, and a two-way mixed model, returning the estimate of ICC and respective confidence intervals (Shrout and Fleiss, 1979; McGraw and Wong, 1996; Revelle, 2012). The ICC is a measure of reproducibility between repeated measurements of the same item, carried out by different observers and can be calculated using the following formula:


$$I\,C\,C=\frac{S_{A}^{2}}{S_{A}^{2}+S_{W}^{2}}$$

with $S_{A}^{2}$ being the variance amongst groups and $S_{W}^{2}$ the variance within groups (Wolak et al., 2012). Having an index going from 0 (no agreement) to 1 (absolute agreement), the ICC value can be interpreted as either poor (<0.50), moderate (0.50 < x < 0.75), good (0.75 < x < 0.90), or excellent (>0.90), when looking at the 95% confidence intervals of the ICC estimate, as suggested by Koo and Li (2016). For the intra-scanner variability, the ICC expresses the fraction of the variance in outcome between individuals, divided by the total variance (Trevethan, 2017). This calculation was carried out separately for the three MRI systems for each of the brain structures mentioned in section “Post processing technique.” In addition, the mean ICC value and confidence intervals were calculated over all brain structures for each MRI system. For the inter-scanner variability, four ICCs were calculated based on absolute agreement, single measurement, and a two-way mixed model, using the mean value of the test and retest scan per MRI system. First, data from all scanners were included, by considering all possible pairwise comparisons. The second, third and fourth ICCs, represented pairwise comparisons between scanners (Ingenia – Achieva, Achieva – GE, and Ingenia – GE). As was done for the intra-scanner measurements, the mean ICC value and confidence intervals were additonally calculated over all brain structures for each MRI system. Taken together, we used the ICC to express the correlation between replicated measurements for the same subject within the same scanner (intra-scanner variability) and in between scanners (inter-scanner variability).

##### Coefficient of variation

Another complementary measure of precision is the CV (%). The CV expresses within-person variability as the ratio of the SD (σ) of repeated measurements divided by their mean (μ), and was calculated by the following formula:


$$CV\left(\%\right)=\left(\frac{\sigma }{\mu }\right)\times 100$$

For the intra-scanner variability, we calculated the CV between the two technical replicates (scan 1 and scan 2) within one person within one scanner [R package: “matrixStats” (rowMeans and rowSds)] (Bengtsson et al., 2021). For the inter-scanner variability, the mean value of the two repeated measurements from each person was taken for each scanner R package “tidyverse” (gather, group_by, summarize). Subsequently, four CVs were calculated. For the first CV, the three mean values of the two repeated measurements from all scanners were considered and the ratio of their SD was divided by the mean. For the second, third and fourth CV, the computations were done in a pairwise manner for each scanner combination (Ingenia – Achieva, Achieva – GE, and Ingenia – GE).

##### Absolute volume differences

Absolute volume differences (mL) between two measurements were also calculated for both intra- and inter-scanner comparisons. For intra-scanner variability, the AVD was calculated as the absolute difference between test (scan one) and retest (scan two) scans within each person within each scanner. For the inter-scanner variability, pairwise differences between scanners were calculated, starting from taking the mean value of the two repeated measurements from each person. The AVD was calculated in a similar way as described previously for the CV, except for the fact that no “all scanner” AVD calculation was carried out, since AVD calculations only allow pairwise comparisons.

##### Dice similarity coefficient

The DSC was calculated to measure the voxel wise overlap between test and retest scan segmentations within each person within each scanner (intra-scanner variability) and for pairwise comparisons between scanners (inter-scanner variability). To this end, one randomly chosen T1w image in a test-retest pair was affinely transformed to the other T1w image in the pair, so that the corresponding brain structure segmentations can be resampled to the same geometric space prior to computing the DSC overlap as:


$$D\,S\,C\,\left(X,Y\right)=\frac{2\,\left(\text{X}\cap \text{Y}\right)}{\left|X\right|+\left|Y\right|}$$

where *X* is the brain structure segmentation from one scan and *Y* is the brain structure segmentation from the other scan after the corresponding spatial transformation. Each of these measures of agreement were computed separately for all brain structure volumes mentioned in subsection “Post processing technique.”

##### Percentual difference

Despite a direct mathematical relationship [factor sqrt (2)] between the percentual difference of two measures and the CV of the same two measurements, percentual differences were reported to interpret reproducibility in the context of yearly atrophy. Lastly, actual volumes were reported to determine the presence of bias between the scanner types.

Significant differences within and between scanners were evaluated for the actual volumes, CV, AVD, and DSC values using a mixed model approach correcting for repeated measurements, with Bonferroni alfa levels of <0.005 [0.05/total number (*n* = 11)] of brain structures. A patient pseudonym (anonymous patient identifier) was included as a random effect to control for the variation in patients, while the scanner pairs (within of between scanner pairs) were included as a fixed effect. Significant differences in actual volumes between the scanner types were evaluated to assess systematic bias between scanner types, while significant differences in measures of agreement (CV, AVD, and DSC) assessed reproducibility.

##### Intra- and inter-scanner variability on patient level

Quantitative measurements and the limits of agreement (LOA) were visualized through Bland–Altman plots using the R package “blandr” to graphically explore individual subject within-scanner measurements as well as to check for possible heteroscedasticity and outliers. Here, the difference between a test and retest scan (*y*-axis) was plotted against the average of the two scans (*x*-axis), including a central horizontal line on the scatter plot depicting the mean difference or “mean bias.” In addition, the SD of the mean bias was used to construct the upper and lower LOA (mean bias ± 1.96 SD). The pre-defined maximum allowed difference was based on *a priori* clinically defined criteria which should not exceed the annual pathological whole brain, GM, and hippocampal atrophy change seen in AD as suggested by Barnes et al. (2009); Sluimer et al. (2008), and Anderson et al. (2012), which is around 2% for larger brain structures and not more than 4.66% for hippocampal volumes. However, it has to be taken into account that atrophy rates are neither spatially nor temporally uniform in MCI and AD patients. If the limits do not exceed the maximum acceptable difference between test and retest scans, and the measurement is not higher than the upper limit of the 95% confidence interval of the upper LOA, nor lower than the lower limit of the 95% CI of the lower LOA, the measurements are considered to be in agreement (Stöckl et al., 2004; Chhapola et al., 2015; Giavarina, 2015).

## Results

### Measurements of Agreement Between Image Pairs at Image Level

Measurements of agreement between image pairs were reported within (intra) and between (inter) scanners to assess agreement at image level (Table 3). There is a very high similarity between different scan-rescan T1w acquired in each scanner, as demonstrated by a reliable affine similarity index, low WM/GM contrast difference, and a maximum scaling factor of 1 for all comparisons between images of the same scanner. Achieva and Ingenia also showed a very reliable affine similarity index. When comparing T1w images of GE and Achieva, the WM/GM contrast showed a higher difference, which could indicate a less consistent segmentation. In fact, when looking at the individual image quality through the absolute CNR values (the CNR value per T1w image, Supplementary Table 1), it is shown that Achieva has a higher contrast than GE.

**TABLE 3:** Pairwise T1w image similarity measures for intra- and inter-scanner comparisons.

Scanner comparison - T1w image	Affine similarity (mean ± SD)	WM/GM contrast (mean ± SD)	Max scale factor (mean ± SD)
**Intra-scanner variability**			
Achieva	0.32 ± 0.02	0.04 ± 0.03	1.00 ± 0.00
Ingenia	0.37 ± 0.03	0.04 ± 0.04	1.00 ± 0.00
GE	0.37 ± 0.03	0.03 ± 0.02	1.00 ± 0.00
**Inter-scanner variability**			
Achieva – Ingenia	0.28 ± 0.01	0.15 ± 0.07	1.01 ± 0.00
Ingenia – GE	0.21 ± 0.02	0.15 ± 0.07	1.01 ± 0.00
Achieva – GE	0.20 ± 0.02	0.30 ± 0.06	1.01 ± 0.00

*The scanner models of the considered image pairs, irrelevant of order, i.e., irrelevant of which image is considered as reference. Affine similarity: mean ± SD of the affine similarity index where >0.2 corresponds to a reliable affine similarity index. WM/GM contrast difference: The absolute difference in WM/GM contrast-to-noise ratio (mean ± SD), with a threshold of acceptability between 0.1 and 0.2. Max scale factor: The mean ± SD of the maximum scaling factor over the three spatial directions, where a value of 1.00 indicates that no scaling is needed, and 1.01 indicates 1% scaling is required. Note that the standard deviation is approximately 0, showing that the scaling needed in pairwise comparisons is subject-independent.*

**FIGURE 1:**
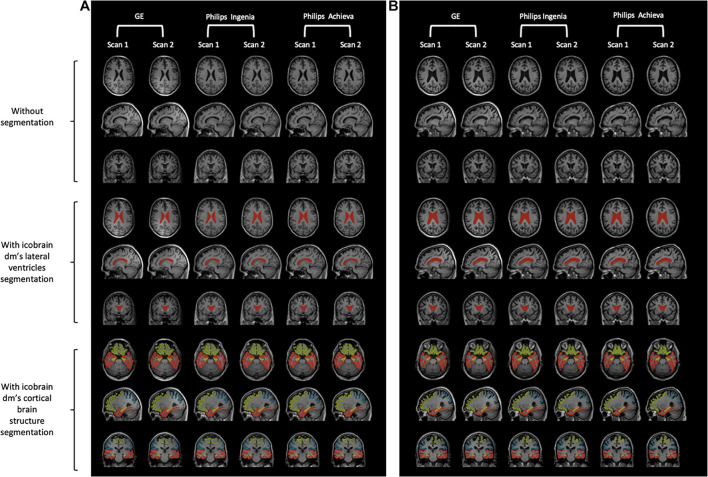
Visual representation of T1w images of each scanner type for a healthy control and a patient with MCI due to AD **(A)** Healthy control. **(B)** MCI due to AD. Both figures: without segmentation (top), with icobrain dm’s segmentation of the lateral ventricles (middle), and with icobrain dm’s segmentation of the cortical brain structures including the hippocampus (bottom). Scanner type (from left to right): “GE Medical Systems Discovery MR750w” – repetition 1, “GE Medical Systems Discovery MR750w” – repetition 2, “Philips Medical Systems Achieva dStream” – repetition 1, “Philips Medical Systems Achieva dStream” – repetition 2, “Philips Medical Systems Ingenia” – repetition 1, “Philips Medical Systems Ingenia” – repetition 2.

The T1w images of each scanner are visualized for two randomly selected subjects in Figure 1. Here, the T1w images of a healthy control (Figure 1A) and a patient with MCI due to AD (Figure 1B) without segmentation (top), with icobrain dm’s segmentation of the LVENT (middle), and with icobrain dm’s segmentation of the cortical brain structures including the hippocampus (bottom) are depicted. These scans and results are shown for both scans on the three different MRI systems.

### Measures of Agreement for Intra- and Inter-Scanner Brain Volumes and Segmentations

Automated volumetric measurements computed by the icobrain dm segmentation software were determined for each MRI scanner, for each of the following brain structures: whole brain, GM, CGM, WM, frontal, parietal and temporal cortices, hippocampal volumes, and LVENT.

#### Intra-Scanner Variability

To examine the reproducibility of measurements within each of the scanners, the CV, AVD, DSC, and the ICC were determined for all previously mentioned brain structures, calculated with icobrain dm (Table 4). Here, the CV expresses the difference between measurements within the same individual, within the same scanner, while the ICC expresses the between-person variance with respect to the total variance.

**TABLE 4:** Intra-scanner variability results per brain structure for three different MRI systems.

Brain structures	CV (%; mean ± SD)	AVD (mL; mean ± SD)	ICC [95% CI]	DSC (mean ± SD)
**Whole brain**				
Achieva	0.18 ± 0.14	3.11 ± 2.50	1.000 [0.999,1.000]	0.92 ± 0.00
Ingenia	0.21 ± 0.14	3.32 ± 2.17	1.000 [0.999,1.000]	0.98 ± 0.00
GE	0.29 ± 0.29	4.70 ± 4.60	0.999 [0.996,1.000]	0.98 ± 0.00
*p-value*	0.418	0.453		0.144
**Gray matter**				
Achieva	0.16 ± 0.12	1.49 ± 1.13	1.000 [0.999,1.000]	0.93 ± 0.01
Ingenia	0.52 ± 0.42	5.07 ± 4.27	0.997 [0.991,0.999]	0.92 ± 0.01
GE	0.43 ± 0.42	3.82 ± 3.62	0.998 [0.995,1.000]	0.93 ± 0.01
*p-value*	0.051	0.045		0.822
**Cortical gray matter**				
Achieva	0.29 ± 0.19	2.61 ± 1.75	0.999 [0.998,1.000]	0.93 ± 0.01
Ingenia	0.54 ± 0.38	4.96 ± 3.74	0.997 [0.992,0.999]	0.92 ± 0.01
GE	0.38 ± 0.48	3.24 ± 3.82	0.998 [0.995,0.999]	0.93 ± 0.02
*p-value*	0.287	0.229		0.893
**White matter**				
Achieva	0.46 ± 0.18	2.97 ± 1.24	0.998 [0.995,0.999]	0.94 ± 0.01
Ingenia	1.06 ± 0.80	6.64 ± 4.61	0.991 [0.971,0.997]	0.94 ± 0.01
GE	0.74 ± 0.34	5.16 ± 2.29	0.994 [0.983,0.998]	0.94 ± 0.01
*p-value*	0.034	0.028		0.088
**Frontal cortex**				
Achieva	0.60 ± 0.35	1.46 ± 0.77	0.998 [0.993,0.999]	0.90 ± 0.01
Ingenia	0.63 ± 0.55	1.52 ± 1.32	0.996 [0.989,0.999]	0.90 ± 0.02
GE	0.99 ± 0.57	2.36 ± 1.41	0.994 [0.982,0.998]	0.89 ± 0.02
*p-value*	0.132	0.164		0.940
**Parietal cortex**				
Achieva	1.58 ± 1.19	2.44 ± 1.69	0.980 [0.936,0.994]	0.87 ± 0.01
Ingenia	1.52 ± 1.24	2.33 ± 1.71	0.981 [0.940,0.994]	**0.87 ± 0.02**
GE	1.78 ± 1.22	2.73 ± 2.17	0.980 [0.938,0.994]	0.87 ± 0.03
*p-value*	0.829	0.850		0.464
**Temporal cortex**				
Achieva	1.94 ± 1.19	3.16 ± 1.88	0.964 [0.890,0.989]	0.89 ± 0.02
Ingenia	1.56 ± 1.30	2.61 ± 2.16	0.974 [0.921,0.992]	0.90 ± 0.01
GE	1.11 ± 0.74	1.82 ± 1.15	0.990 [0.970,0.997]	0.90 ± 0.02
*p-value*	0.148	0.151		0.165
**Hippocampus, total**				
Achieva	1.44 ± 0.87	0.14 ± 0.09	0.986 [0.957,0.996]	0.90 ± 0.01^∧^
Ingenia	1.63 ± 0.91	0.16 ± 0.08	0.985 [0.953,0.995]	0.91 ± 0.01^•^
GE	0.95 ± 0.67	0.09 ± 0.06	0.994 [0.981,0.998]	0.93 ± 0.01^∧•^
*p-value*	0.142	0.119		**<0.001**
**Hippocampus, left**				
Achieva	2.90 ± 1.87	0.14 ± 0.09	0.956 [0.865,0.986]	0.90 ± 0.01^∧^
Ingenia	**3.14 ± 2.15**	0.15 ± 0.10	**0.941 [0.823,0.981]**	0.90 ± 0.02^•^
GE	1.46 ± 1.14	0.07 ± 0.05	0.988 [0.963,0.996]	0.92 ± 0.01^∧•^
*p-value*	0.049	0.063		**<0.001**
**Hippocampus, right**				
Achieva	1.35 ± 1.45	0.06 ± 0.06	0.988 [0.963,0.996]	0.91 ± 0.01^∧^
Ingenia	1.11 ± 0.88	0.05 ± 0.04	0.992 [0.974,0.997]	0.91 ± 0.01
GE	1.16 ± 0.71	0.06 ± 0.04	0.992 [0.973,0.997]	0.93 ± 0.01^∧^
*p-value*	0.848	0.957		**<0.001**
**Lateral ventricles**				
Achieva	0.66 ± 0.84	0.28 ± 0.29	0.999 [0.998,1.000]	0.96 ± 0.01
Ingenia	0.75 ± 1.09	0.34 ± 0.46	0.999 [0.997,1.000]	0.96 ± 0.01
GE	1.13 ± 1.33	0.55 ± 0.78	0.997 [0.973,0.997]	0.97 ± 0.01
*p-value*	0.407	0.475		0.054

**All volumes**	**Mean CV (%; mean ± SD)**	**Mean AVD (mL; mean ± SD)**	**Mean ICC [95% CI]**	**Mean DSC (mean ± SD)**

Achieva	1.05 ± 0.87	1.63 ± 1.29	0.988 [0.977,0.998]	0.91 ± 0.01
Ingenia	1.15 ± 0.81	2.47 ± 2.30	0.987 [0.975,0.998]	0.92 ± 0.01
GE	0.95 ± 0.46	2.24 ± 1.88	0.993 [0.989,0.997]	0.93 ± 0.04

*Brain volumes used to calculate measurements of precision were computed by the icobrain dm segmentation software. Coefficients of variation (CV, %), AVD (mL), intraclass correlation coefficients (ICC, [95% CI]), and Dice similarity coefficients (DSC, mean ± SD) were reported. The highest CV and lowest ICC and DSC values amongst all structures were highlighted in “bold.” Achieva: Philips Medical Systems Achieva dStream 1.5T. Ingenia: Philips Medical Systems Ingenia 3T. GE: GE Discovery MR750w 3T. *p*-value < 0.005 for “all scanners” differences were highlighted in “bold.” ^∧^*p*-value < 0.005 between Achieva and GE ^⋅^*p*-value < 0.005 between Ingenia and GE.*

The individual CV values (mean ± SD) were between 0.16 ± 0.12 and 3.14 ± 2.15%. The intra-scanner CVs over all volumes were similar on the three scanners, with 1.05 ± 0.87% for Achieva, 1.15 ± 0.81% for Ingenia and 0.95 ± 0.46% for GE. The AVDs and ICCs showed the same trend as the CV values. The ICC showed no scores below (mean [CI]) 0.941 [0.823,0.981] (HIP-L, Ingenia). The ICC scores tended to decrease slightly when looking at smaller regional brain volumes such as the hippocampal volumes, except for the right hippocampus. The DSC values (mean ± SD) went from 0.87 ± 0.02 (PC, Ingenia) to 0.98 ± 0.00 (WB, GE). The intra-scanner DSC (mean ± SD) overall volumes were 0.91 ± 0.01 for Achieva, 0.92 ± 0.01 for Ingenia and 0.93 ± 0.04 for GE. Significant differences in DSC values were reported for the hippocampal volumes (*p* < 0.001). The estimated effect, standard error, *z*-values, and adjusted *p*-values (Bonferroni) per pairwise differences for each brain structure showing a significant overall difference in DSC values are reported in Supplementary Table 2.

#### Inter-Scanner Variability

To examine the inter-scanner variability, the AVD, CV, DSC, and ICC and were determined for Achieva – Ingenia, Achieva – GE, Ingenia – GE, and an all-scanner comparison (Table 5). Here, the CV expresses the differences between the three MRI systems, while the ICC was used to express how similar the observations are across the three MRI systems.

**TABLE 5:** Inter-scanner variability results per brain structure for three different MRI systems.

Brain structures	CV (mean ± SD, in %)				ICC [95% CI]			
	All scanners	Ingenia – Achieva	Achieva – GE	Ingenia – GE	All scanners	Ingenia – Achieva	Achieva – GE	Ingenia – GE
Whole brain	0.53 ± 0.27	0.49 ± 0.43	0.36 ± 0.23	0.58 ± 0.35	0.999 [0.997,0.999]	0.998 [0.995, 0.999]	0.999 [0.996, 1.000]	0.999 [0.996, 0.999]
Gray matter	2.88 ± 1.30	0.44 ± 0.62	3.73 ± 1.73	3.30 ± 1.59	0.990 [0.976,0.996]	0.998 [0.993, 0.999]	0.985 [0.954, 0.995]	0.985 [0.954, 0.995]
Cortical gray matter	2.69 ± 1.18	0.57 ± 0.62	3.49 ± 1.64	3.02 ± 1.27	0.992 [0.981,0.997]	0.997 [0.991, 0.999]	0.988 [0.961, 0.996]	0.988 [0.961, 0.996]
White matter	4.75 ± 1.76	0.76 ± 0.43	5.46 ± 1.91	**5.93 ± 2.31**	0.972 [0.934, 0.990]	0.995 [0.985,0.999]	0.971 [0.910, 0.991]	**0.961 [0.901, 0.991]**
Frontal cortex	3.71 ± 1.38	0.49 ± 0.39	4.72 ± 1.75	4.39 ± 1.76	0.992 [0.981, 0.997]	0.998 [0.994, 0.999]	0.989 [0.966, 0.997]	0.989 [0.966, 0.997]
Parietal cortex	3.85 ± 1.98	0.74 ± 0.37	4.70 ± 2.62	4.65 ± 2.60	0.983 [0.959, 0.994]	0.996 [0.987, 0.999]	0.978 [0.932, 0.993]	0.978 [0.932, 0.993]
Temporal cortex	1.77 ± 1.04	1.42 ± 0.98	2.29 ± 1.56	1.22 ± 0.74	0.984 [0.961, 0.995]	0.989 [0.964, 0.996]	0.972 [0.915, 0.991]	0.972 [0.915, 0.991]
Hippocampus, total	1.64 ± 0.90	1.14 ± 0.94	1.52 ± 0.85	1.80 ± 1.48	0.986 [0.959, 0.996]	0.992 [0.973, 0.997]	0.987 [0.961, 0.996]	0.988 [0.961, 0.996]
Hippocampus, left	2.64 ± 1.79	2.08 ± 1.92	2.68 ± 1.57	2.43 ± 2.74	0.968 [0.924, 0.989]	0.968 [0.902, 0.990]	0.980 [0.940, 0.994]	0.980 [0.940, 0.993]
Hippocampus, right	2.12 ± 1.37	1.16 ± 1.18	2.27 ± 1.62	2.16 ± 2.11	0.979 [0.945, 0.993]	0.989 [0.967, 0.997]	0.976 [0.925, 0.992]	0.975 [0.925, 0.992]
Lateral ventricles	1.45 ± 0.98	1.54 ± 1.28	0.70 ± 0.76	1.56 ± 1.29	0.997 [0.994, 0.999]	0.996 [0.988, 0.999]	0.999 [0.998, 1.000]	0.999 [0.998, 0.999]

All volumes	**Mean CV (mean ± SD, in %)**				**Mean ICC [95% CI]**			
	
	2.55 ± 1.22	0.99 ± 0.53	2.90 ± 1.68	2.82 ± 1.63	0.986 [0.979, 0.992]	0.992 [0.986, 0.998]	0.984 [0.977, 0.990]	0.983 [0.990, 0.975]

**Brain structures**	**AVD (mean ± SD, in mL)**				**DSC (mean ± SD)**			
	**All scanners**	**Ingenia – Achieva**	**Achieva – GE**	**Ingenia – GE**	**All scanners**	**Ingenia – Achieva**	**Achieva – GE**	**Ingenia – GE**

Whole brain	–	7.91 ± 6.66	5.80 ± 3.41	9.56 ± 5.97	0.97 ± 0.01	0.98 ± 0.00	0.97 ± 0.00	0.97 ± 0.00
Gray matter	–	4.12 ± 5.35	33.91 ± 14.45	30.03 ± 12.56	0.89 ± 0.02	0.91 ± 0.01	0.88 ± 0.02	0.89 ± 0.02
Cortical gray matter	–	5.12 ± 5.18	30.24 ± 12.95	26.16 ± 9.77	0.90 ± 0.02	0.92 ± 0.01	0.88 ± 0.02	0.89 ± 0.02
White matter	–	4.97 ± 3.01	36.35 ± 3.01	**39.58 ± 3.00**	0.92 ± 0.01	0.93 ± 0.01	0.91 ± 0.01	0.92 ± 0.01
Frontal cortex	–	1.30 ± 1.17	11.35 ± 3.63	10.42 ± 3.28	0.86 ± 0.02	0.89 ± 0.01	0.84 ± 0.01	0.85 ± 0.01
Parietal cortex	–	1.19 ± 0.63	6.74 ± 3.29	0.67 ± 3.33	0.82 ± 0.03	0.85 ± 0.02	**0.80 ± 0.02**	0.82 ± 0.02
Temporal cortex	–	2.24 ± 1.49	3.59 ± 2.39	1.93 ± 1.12	0.87 ± 0.02	0.89 ± 0.01	0.86 ± 0.02	0.86 ± 0.02
Hippocampus, total	–	0.10 ± 0.06	0.14 ± 0.06	0.17 ± 0.12	0.90 ± 0.01	0.91 ± 0.01	0.89 ± 0.01	0.90 ± 0.01
Hippocampus, left	–	0.09 ± 0.09	0.13 ± 0.07	0.11 ± 0.11	0.89 ± 0.02	0.90 ± 0.01	0.89 ± 0.01	0.89 ± 0.01
Hippocampus, right	–	0.05 ± 0.06	0.11 ± 0.08	0.11 ± 0.10	0.90 ± 0.01	0.91 ± 0.01	0.90 ± 0.01	0.90 ± 0.01
Lateral ventricles	–	0.86 ± 0.99	0.32 ± 0.31	0.90 ± 0.90	0.95 ± 0.01	0.96 ± 0.01	0.95 ± 0.01	0.95 ± 0.01

All volumes	**Mean AVD (mean ± SD, in mL)**				**Mean DSC (mean ± SD)**			
	–	2.55 ± 2.61	11.70 ± 14.49	10.88 ± 14.34	0.90 ± 0.04	0.91 ± 0.03	0.89 ± 0.05	0.89 ± 0.04

*Brain volumes used to calculate measurements of precision were computed by the icobrain dm segmentation software. Coefficient of variation (CV, %), intraclass correlation coefficients (ICC, [95% CI]), absolute volume differences (AVD, mL) and Dice similarity coefficients (DSC, mean ± SD) was reported for all pairwise comparisons and three-scanner comparisons (“All scanners”). The highest CV and lowest ICC and DSC values amongst all structures were highlighted in **bold**. Achieva: Philips Medical Systems Achieva dStream 1.5T. Ingenia: Philips Medical Systems Ingenia 3T. GE: GE Discovery MR750w 3T.*

The individual CV values (mean ± SD) were between 0.36 ± 0.23 (WB, Achieva – GE) and 5.93 ± 2.31% (WM, Ingenia – GE). The inter-scanner CV over all volumes were on average 2.55 ± 1.22% for all-scanner comparisons, 0.99 ± 0.53% for Ingenia – Achieva, 2.90 ± 1.68% for Achieva – GE, and 2.82 ± 1.63% for Ingenia – GE. The AVDs and ICCs showed the same trend as the CV values, with the ICC showing no mean scores below 0.961 [0.901, 0.991] (WM, Ingenia – GE). The DSC values (mean ± SD) were in between 0.80 ± 0.02 (WB, Achieva – GE) and 0.98 ± 0.00 (WB, Ingenia – Achieva). The inter-scanner DSCs (mean ± SD) over all volumes were 0.90 ± 0.04 for all-scanner comparisons, 0.91 ± 0.03 for Ingenia – Achieva, 0.89 ± 0.05 for Achieva – GE, and 0.89 ± 0.04 for Ingenia – GE.

#### Individual Quantitative Intra- and Inter-Scanner Variability

**FIGURE 2:**
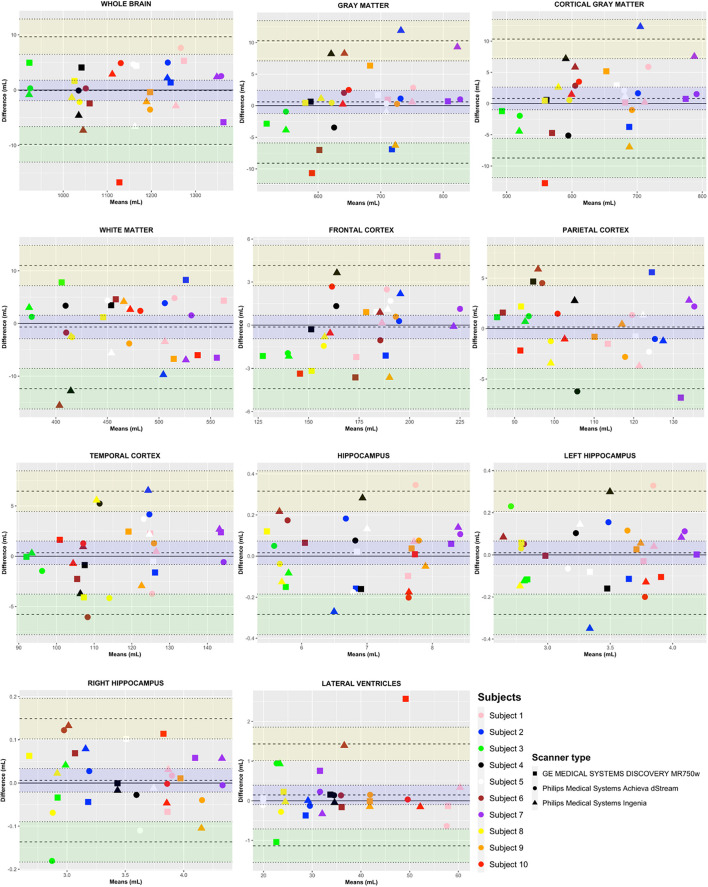
Bland–Altman plots for individual brain structures. Variability results (intra- and inter-scanner, as well as intra- and inter-subject variability) presented in a Bland–Altman plot per brain structure for three different MRI systems; Philips Medical Systems Achieva dStream 1.5T, Philips Medical Systems Ingenia 3T, and GE Discovery MR750w 3T, computed by icobrain dm. The *y*-axis represents the difference in mL [test (scan 1) – retest (scan 2)]. The *x*-axis represents the mean in mL of scan 1 and scan 2 [(scan 1 + scan 2)/2]. The quantitative measurements are presented per subject (color-coded differentiation) and per scanner (symbol-coded differentiation). Here, the limit of agreement (LOA, upper LOA in yellow and lower LOA in green) represents the 95% prediction interval [1.96 SD].

The quantitative measurements computed by icobrain dm for the test (scan 1) and retest (scan 2) scans per subject (color-coded differentiation) and per scanner (symbol-coded differentiation) were visually (Figure 2) and statistically (Supplementary Table 3) presented using a Bland–Altman plot to detect possible deficiencies in individual reliability, heteroscedasticity, and outliers.

The *Y*-coordinate of a point shows the difference in mL between scan one and scan two, while the *X*-coordinate indicates the mean between the two volumes. By showing the results of the three different scanners for each person separately (with the three different plot characters according to the scanner), we depicted the inter-scanner variability per subject between the different scanners.

The within-subject inter-scanner-variability is visible in the *X*-axis direction when looking at the inter-scanner means in mL [(scan one + scan two)/2] differences. Furthermore, between-subject variation is visible in the *X*-axis direction, looking at the difference in means between individuals. Here, the LOA represents the 95% prediction interval [1.96 SD], where a smaller range indicates a better agreement.

According to the Bland–Altman plot, the GE result falls outside of the LLOA for subject 10 for WB and CGM, and outside of the ULOA for LVENT. To identify if there is an underlying reason behind this larger intra-scanner variability, a double-check of the native MRI sequences was performed for this specific subject 10. Evaluation by a neuroradiologist (GA) revealed no significant MRI artifacts. Both acquisitions had a similar gray and WM contrast. However, evaluation of icobrain dm’s segmentation revealed a slight oversegmentation of the cortex in the superior sagittal sinus, which might be a partial explanation for the increased difference between the two scans. Furthermore, no heteroscedasticity nor a specific pattern regarding intra- or inter-scanner variability was found for any of the regions of interest.

#### Percentual Differences

Percentual differences are reported in Table 6. When looking at the intra-scanner variability results, the largest percentual volume difference was seen for the left hippocampus (mean ± SD, 4.47 ± 3.13, and Ingenia), while pairwise comparisons showed the largest difference for WM (mean ± SD, 8.74 ± 3.47, and Ingenia – GE). These findings were in line with the intra-CV and intra-ICC values. The smallest percentual volume difference was found for gray matter for intra-scanner (mean ± SD, 0.22 ± 0.17, Achieva) and whole brain for inter-scanner variability results (mean ± SD, 0.52 ± 0.32, Achieva – GE).

**TABLE 6:** Percentual volume differences per brain structure.

Brain structures	Percentage (mean ± SD, in %)							
	Intra-scanner				Inter-scanner			
	Achieva	Ingenia	GE	All intra-scanner	All scanners	Ingenia – Achieva	Achieva – GE	Ingenia – GE
Whole brain	0.26 ± 0.20	0.30 ± 0.21	0.42 ± 0.41	0.32 ± 0.28	–	0.69 ± 0.60	0.52 ± 0.32	0.82 ± 0.49
Gray matter	0.22 ± 0.17	0.73 ± 0.59	0.61 ± 0.60	0.52 ± 0.53	–	0.63 ± 0.86	5.11 ± 2.34	4.52 ± 2.01
Cortical gray matter	0.41 ± 0.27	0.76 ± 0.53	0.55 ± 0.69	0.57 ± 0.53	–	0.81 ± 0.87	4.79 ± 2.22	4.14 ± 1.71
White matter	0.65 ± 0.25	1.51 ± 1.16	1.05 ± 0.48	1.06 ± 0.79	–	1.08 ± 0.60	8.06 ± 2.92	**8.74 ± 3.47**
Frontal cortex	0.85 ± 0.49	0.88 ± 0.77	1.41 ± 0.81	1.05 ± 0.72	–	0.68 ± 0.55	6.43 ± 2.32	5.98 ± 2.34
Parietal cortex	2.48 ± 1.71	2.14 ± 1.72	2.52 ± 1.72	2.17 ± 1.58	–	1.04 ± 0.53	6.38 ± 3.49	6.31 ± 3.47
Temporal cortex	2.75 ± 1.71	2.19 ± 1.80	1.57 ± 1.07	2.31 ± 1.67	–	1.98 ± 1.35	3.17 ± 2.14	1.68 ± 0.99
Hippocampus, total	2.02 ± 1.22	2.31 ± 1.29	1.35 ± 0.95	1.90 ± 1.20	–	2.16 ± 1.22	2.16 ± 1.22	2.56 ± 2.10
Hippocampus, left	4.02 ± 2.52	**4.47 ± 3.13**	2.09 ± 1.65	3.53 ± 2.64	–	2.97 ± 2.77	3.88 ± 2.32	3.47 ± 3.87
Hippocampus, right	1.92 ± 2.09	1.56 ± 1.22	1.63 ± 0.99	1.71 ± 1.46	–	1.65 ± 1.66	3.15 ± 2.20	3.03 ± 2.91
Lateral ventricles	0.93 ± 1.16	1.04 ± 1.49	1.59 ± 1.88	1.20 ± 1.53	–	2.22 ± 2.21	0.99 ± 0.99	2.23 ± 2.23
All volumes	1.48 ± 1.21	1.63 ± 1.16	1.34 ± 0.65	1.48 ± 1.01	–	1.45 ± 0.79	4.06 ± 2.36	3.95 ± 2.32

*Percentual volume difference [Percentage (%), mean ± SD] for each individual brain volume. All intra scanner volumes represent the mean percentual volume difference of all three scanner types combined, since percentual volume differences only allow pairwise comparisons. The largest percentual volume differences (intra- and inter-scanner variability) were highlighted in **bold**.*

#### Actual Brain Structure Volumes

Actual volumes for all brain structures were reported as mean ± SD (Table 7). To assess systematic bias between the three MRI systems, a mixed model approach correcting for repeated measures with post-hoc Bonferroni correction was employed. Intra-scanner variability results showed no significant within-scanner differences for any of the brain structure volumes. For Achieva – Ingenia, whole brain and LVENT were significantly different (*p* < 0.001). For Achieva – GE, GM, CGM, WM, frontal, parietal, and temporal cortices, as well as the right hippocampus showed significant differences (p < 0.001). Significant differences for all brain volumes (p < 0.001), except the TC, total, and left hippocampus, were found for Ingenia – GE. The estimated effect, standard error, *z*-values and adjusted *p*-values (Bonferroni) per pairwise differences for each brain structure showing a significant overall difference in actual volumes are reported in Supplementary Table 4.

**TABLE 7:** Actual volumes per brain structure for all three MRI systems.

Brain structures	Volumes (mean ± SD, in mL)				
	Achieva	Ingenia	GE	All scanners	*p-value*
Whole brain	1139.77 ± 130.19^#^	1132.64 ± 129.54^#•^	1142.20 ± 132.82^•^	1138.20 ± 126.33	<0.001
Gray matter	683.08 ± 80.55^∧^	679.19 ± 81.76^•^	649.16 ± 87.20^∧•^	670.47 ± 81.77	<0.001
Cortical gray matter	650.81 ± 79.06^∧^	646.72 ± 80.56^•^	620.57 ± 85.32^∧•^	639.37 ± 79.99	<0.001
White matter	456.69 ± 52.48^∧^	453.45 ± 50.43^•^	493.04 ± 53.18^∧•^	467.73 ± 53.43	<0.001
Frontal cortex	180.02 ± 24.40^∧^	179.09 ± 23.39^•^	168.67 ± 24.96^∧•^	175.93 ± 23.99	<0.001
Parietal cortex	111.81 ± 14.35^∧^	111.75 ± 14.45^•^	105.07 ± 17.05^∧•^	109.54 ± 15.14	<0.001
Temporal cortex	118.04 ± 13.45^∧^	116.31 ± 14.46	115.25 ± 15.29^∧^	116.54 ± 13.97	<0.001
Hippocampus, total	6.89 ± 1.00	6.92 ± 0.99	6.92 ± 0.93	6.91 ± 0.94	0.716
Hippocampus, left	3.35 ± 0.49	3.38 ± 0.50	3.46 ± 0.47	3.40 ± 0.47	0.006
Hippocampus, right	3.54 ± 0.54^∧^	3.55 ± 0.51^•^	3.45 ± 0.48^∧•^	3.51 ± 0.49	<0.001
Lateral ventricles	34.67 ± 12.13^#^	35.44 ± 12.91^#•^	34.55 ± 12.12^•^	34.89 ± 11.96	<0.001

*Actual volumes (Volumes, mL) computed by icobrain dm were reported as mean ± SD. Achieva: Philips Medical Systems Achieva dStream 1.5T. Ingenia: Philips Medical Systems Ingenia 3T. GE: GE Discovery MR750w 3T. *p*-value: Difference between the three scanner types. *Post-hoc* comparison: ^∧^*p*-value < 0.005 between Achieva and GE. ^•^*p*-value < 0.005 between Ingenia and GE. ^#^*p*-value < 0.005 between Achieva and Ingenia.*

## Discussion

As the potential added diagnostic value of AI-based automated volumetry on brain MRI scans might at least in part be neutralized by intra- and inter-scanner variability, a thorough evaluation of the measurement error and variability in clinical routine circumstances is crucial. In the current study, the intra- and inter-scanner variability of global, cortical, and subcortical brain volumes was evaluated using the CE marked and FDA cleared icobrain dm software on three different MRI systems.

It is known that intra-scanner variability exists and depends on several uncontrollable (short-term physiological fluctuations), semi-controllable (head motion, subject-positioning, noise, and measurement error) and controllable (impact of day-to-day, time of day, and medication) factors. Previous studies have reported a time-of-day dependence of MRI-based global brain volume calculations (Trefler et al., 2016; Dieleman et al., 2017). In this study, all patients were scanned in the morning, eliminating both the impact of day-to-day and morning/night differences as additional variables. Subject-positioning variation was minimized, by placing the subject in the MRI scanner by the same operator, through a standardized procedure. Nevertheless, when comparing brain structure segmentations of repeated (intra- or inter-scanner) scans using Dice overlap, affine image alignment and resampling was still required. This post-processing step typically leads to an additional variability, which is an unavoidable limitation of this type of agreement measurement between repeated scans.

Overall, low intra-scanner variability of the MRI measures was found. The ICC showed no scores below (mean [CI]) 0.941 [0.823, 0.981] (HIP-L, Ingenia), indicating a good intra-scanner agreement for all intra-scanner comparisons. The largest intra-scanner variability was observed in the left hippocampus, which can be explained by the smaller size of this brain structure (Struyfs et al., 2020) and the complexity of its delineation. In addition, a higher variability compared to other larger brain regions was demonstrated for the LVENT, related to the CSF presence and existing short-term physiological fluctuations (Dieleman et al., 2017). Similar to the intra-scanner variability, a low inter-scanner variability of the icobrain dm measures was observed. The ICC showed no mean scores below 0.961 [0.901, 0.991] (WM, Ingenia – GE). In addition, significant differences between the actual volumes and the visual assessment of Bland–Altman plots did not reveal any systematic pattern regarding intra or inter-scanner bias. The mixed modeling approach showed that the significance came from the DSC measures and actual volumes, while CV and AVD differences were not statistically significant. This might indicate that DSC are more sensitive than volumetric criteria, since the overlap between two segmentations is assessed, while an imperfect overlap might be compensated for when calculating volumes.

However, statistical significance and clinical relevance should not be mistakenly conflated since one does not necessarily imply the other. Previous studies based on longitudinal data have reported annual rates of atrophy [in % of atrophy/year, mean (95% CIs)] for several brain volumes, including whole brain [0.32% (0.10–0.54)], temporal lobes [0.68% (0.42–0.93)], hippocampi [0.82% (0.53–1.11)], and LVENT [650 mm^3^/y (333–968)] due to normal aging (Good et al., 2001; Scahill et al., 2003; Biberacher et al., 2016; Schippling et al., 2017; Vinke et al., 2018). Annual atrophy rates of around 2% have been observed in Alzheimer’s patients for whole brain (Sluimer et al., 2008) and GM volumes (Anderson et al., 2012). In addition, a meta-analysis on hippocampal atrophy rates in AD patients and controls reported annualized hippocampal atrophy rates of 4.66% (3.92–5.40) for AD patients, while an atrophy rate of 1.41% (0.52–2.30) was reported for healthy individuals (Barnes et al., 2009). According to our study, the within-scanner difference in percentage for whole brain volumes, taken within a time span of 3 h, are similar to the previously reported annual volume decline for healthy individuals, although a pathologic whole brain volume change, as seen in AD, would go beyond the observed intra- and inter-scanner measurement error. In the light of these events, attention needs to be paid when comparing MRI scans obtained with different protocols, since even with the same vendor, harmonized protocols, and elimination of the previously mentioned controllable influencing factors, a volumetric bias remains. On the other hand, our volumetric analysis was performed in a “cross-sectional” way, where each individual scan was segmented independently. It is known that “longitudinal” methods, which simultaneously analyze two or more brain scans, have a significantly lower measurement error, and should be preferred over cross-sectional measurements, for computing atrophy of brain structures.

### Harmonization

The three MRI systems that were used in this study had a difference in coils and channels per coil between the systems (16 channels vs. 32 channels), resulting in differences in the signal-to-noise (SNR) ratio. In this study design we used coils that were directly purchased from the manufacturer. In addition, the difference in FOV between the three MRI systems can be explained by the fact that there was no phase oversampling available for the GE. To compensate for this, a large FOV was employed, enabling the acquirement of the same resolution and number of phase-encoded lines as for the other vendors, and hence SNR was not affected. Furthermore, the reconstruction resolution for GE was slightly lower compared to the other MRI systems, as this could not be chosen freely and interpolation factors > 2 were avoided. Nevertheless, the in-plane image resolution of 1 mm × 1 mm is well suited for brain segmentation. The difference in image resolution could have a slight effect on the segmentation of some regions which contain a lot of complicated borders. The total duration of the scan was also larger for GE than for the other systems, since GE does not offer TFE sequence, but has its own BRAVO sequence, a TFE sequence that is optimized for brain recording. The disadvantage, in our case, is that the adjustable parameters are limited, including control over the scan duration. Another potential cause of variability that was not investigated in this study, are the scanner-specific differences in post processing of the raw data. For example, the GE scanner that was utilized in this study ended up with 280 instead of 288 slices (as with Achieva and Ingenia). This was the consequence of an implicit oversampling and the “throwing away” of the outer slices during the reconstruction, which is GE specific. Additional efforts are needed to deepen our understanding of the effects on inter-scanner variability of these scanner-specific post processing differences.

An additional limitation of this study was the small sample size (*n* = 10) that was not sufficient to draw realistic conclusions regarding disease related variability, but, however, producing a total of 60 MRI scans which allowed for the analysis of within-subject differences. In addition, since only one automated volumetric software tool was utilized in this study, it would be beneficial to investigate the effect of different automated volumetric software’s on the intra and inter-variability across different MRI systems.

Follow-up of brain MRI scans can aid in tracking disease progression, which may be relevant for research purposes. In addition, MRI can display the presence of typical brain atrophy patterns correlated to specific neurodegenerative diseases. Analyzing and subsequently improving intra- and inter-scanner variability can bring us closer to comparing MRI scans from the same individual, taken from different centers. Being able to compare multi-center MRI scans is also useful in clinical trials, where MRI scans from different scanners can then be pooled for data analysis. Harmonizing inter-center MRI scans might aid multi-center research, but its application in a clinical setting remains challenging. Therefore, techniques which allow for the harmonization of MRI data, e.g., based on AI, would be very valuable to overcome these obstacles. This approach might allow comparison of recent MRI scans with older MRI scans (using different acquisition techniques) over a longer period.

In conclusion, harmonized acquisition sequences were able to produce good quality brain scans on different MRI scanners and were suitable for automated brain segmentation. In addition, observed intra- and inter-scanner measurement error was smaller than the annual pathologic whole brain volume change, as seen in AD. Harmonized scans obtained with different scanners of the same manufacturer had a measurement error closer to the intra-scanner performance. The gap between intra- and inter-scanner comparisons grew when comparing scans from different manufacturers. This was observed at image level in terms of image contrast, image similarity and geometry, and translated into a higher variability of automated brain volumetry. However, on average, intra and inter-scanner variability results showed a good overlap of brain structure segmentation (mean DSC > 0.88) and good reproducibility within- (mean CV < 2%) and between-scanners (mean CV < 5%) was obtained over global, cortical, and subcortical brain structures.

## Data Availability Statement

All data are available from the corresponding author on reasonable request.

## Ethics Statement

The studies involving human participants were reviewed and approved by this randomized prospective study was approved by the Ethical Committee of UZ Brussel in Brussels, Belgium (Reference nr: 2020-079). Written informed consent of all participants and/or legal representatives (in case of dementia) was obtained. The patients/participants provided their written informed consent to participate in this study.

## Author Contributions

G-JA: conceptualization, investigation, resources, data curation, and writing – original draft. MW: conceptualization, investigation, resources, data curation, formal analysis, validation, visualization, and writing – original draft. DMS: methodology, software, validation, formal analysis, and writing – review and editing. MN: data curation, and writing – review and editing. TV: writing – review and editing. A-MV, YD, and GN: conceptualization. NB: conceptualization, and writing – review and editing. HR: methodology, resources, supervision, and validation. EF: formal analysis, and writing – review and editing. DS: conceptualization and software. WH: conceptualization, validation, and writing – review and editing. MB: supervision, and writing – review and editing. JM: conceptualization, resources, supervision, funding acquisition, and writing – review and editing. SE: conceptualization, resources, supervision, project administration, funding acquisition, and writing – review and editing. All authors critically revised and approved the content of the final manuscript before submission.

## Conflict of Interest

DMS, DS, and WH are employed by icometrix. SE serves as a consultant for icometrix, and served as consultant for Biogen, Danone, Eisiai, Novartis, Nutricia, Pfizer, and Roche. The remaining authors declare that the research was conducted in the absence of any commercial or financial relationships that could be construed as a potential conflict of interest.

## Publisher’s Note

All claims expressed in this article are solely those of the authors and do not necessarily represent those of their affiliated organizations, or those of the publisher, the editors and the reviewers. Any product that may be evaluated in this article, or claim that may be made by its manufacturer, is not guaranteed or endorsed by the publisher.

## References

[B1] Albert M. S. DeKosky S. T. Dickson D. Dubois B. Feldman H. H. Fox N. C. (2011). The diagnosis of mild cognitive impairment due to Alzheimer’s disease: recommendations from the National Institute on Aging-Alzheimer’s Association workgroups on diagnostic guidelines for Alzheimer’s disease. *Alzheimers Dement.* 7 270–279. 10.1016/j.jalz.2011.03.008 21514249 PMC3312027

[B2] Anderson V. M. Schott J. M. Bartlett J. W. Leung K. W. Miller D. H. Fox N. C. (2012). Gray matter atrophy rate as a marker of disease progression in AD. *Neurobiol. Aging* 33 1194–1202. 21163551 10.1016/j.neurobiolaging.2010.11.001 PMC3657171

[B3] Apostolova L. G. Green A. E. Babakchanian S. Hwang K. S. Chou Y. Y. Toga A. W. (2012). Hippocampal atrophy and ventricular enlargement in normal aging, mild cognitive impairment (MCI), and Alzheimer disease. *Alzheimer Dis. Assoc. Disord.* 26 17–27. 10.1097/WAD.0b013e3182163b62 22343374 PMC3286134

[B4] Barnes J. Bartlett J. W. van de Pol L. A. Loy C. T. Scahill R. I. Frost C. (2009). A meta-analysis of hippocampal atrophy rates in Alzheimer’s disease. *Neurobiol. Aging* 30 1711–1723. 10.1016/j.neurobiolaging.2008.01.010 18346820 PMC2773132

[B5] Bengtsson H. Ahlmann-Eltze C. Corrada Bravo H. Gentleman R. Gleixner J. Hickey P. (2021). Functions that Apply to Rows and Columns of Matrices (and to Vectors). *Packag. “matrixStats”* 0.60.1. Available at: https://github.com/HenrikBengtsson/matrixStats (Accessed August 24, 2021).

[B6] Biberacher V. Schmidt P. Keshavan A. Boucard C. C. Righart R. Sämann P. (2016). Intra- and interscanner variability of magnetic resonance imaging based volumetry in multiple sclerosis. *Neuroimage* 142 188–197. 10.1016/j.neuroimage.2016.07.035 27431758

[B7] Cajanus A. Solje E. Koikkalainen J. Lötjönen J. Suhonen N. M. Hallikainen I. (2019). The association between distinct frontal brain volumes and behavioral symptoms in mild cognitive impairment, Alzheimer’s disease, and frontotemporal dementia. *Front. Neurol.* 10:1059. 10.3389/fneur.2019.01059 31632342 PMC6786130

[B8] Chhapola V. Kanwal S. K. Brar R. (2015). Reporting standards for Bland–Altman agreement analysis in laboratory research: a cross-sectional survey of current practice. *Ann. Clin. Biochem.* 52 382–386. 10.1177/0004563214553438 25214637

[B9] Clerx L. van Rossum I. A. Burns L. Knol D. L. Scheltens P. Verhey F. (2013). Measurements of medial temporal lobe atrophy for prediction of Alzheimer’s disease in subjects with mild cognitive impairment. *Neurobiol. Aging* 34 2003–2013. 10.1016/j.neurobiolaging.2013.02.002 23540941

[B10] Den Heijer T. Van Der Lijn F. Koudstaal P. J. Hofman A. Van Der Lugt A. Krestin G. P. (2010). A 10-year follow-up of hippocampal volume on magnetic resonance imaging in early dementia and cognitive decline. *Brain* 133 1163–1172. 10.1093/brain/awq048 20375138

[B11] Dieleman N. Koek H. L. Hendrikse J. (2017). Short-term mechanisms influencing volumetric brain dynamics. *Neuroimage Clin.* 16 507–513. 10.1016/j.nicl.2017.09.002 28971004 PMC5609861

[B12] Duara R. Loewenstein D. A. Potter E. Appel J. Greig M. T. Urs R. (2008). Medial temporal lobe atrophy on MRI scans and the diagnosis of Alzheimer disease. *Neurology* 71 1986–1992. 10.1212/01.wnl.0000336925.79704.9f 19064880 PMC2676975

[B13] Dubois B. Feldman H. H. Jacova C. Hampel H. Molinuevo J. L. Blennow K. (2014). Advancing research diagnostic criteria for Alzheimer’s disease: the IWG-2 criteria. *Lancet Neurol.* 13 614–629. 10.1016/S1474-4422(14)70090-0 24849862

[B14] Ferrarini L. Palm W. M. Olofsen H. van Buchem M. A. Reiber J. H. C. Admiraal-Behloul F. (2006). Shape differences of the brain ventricles in Alzheimer’s disease. *Neuroimage* 32 1060–1069. 10.1016/j.neuroimage.2006.05.048 16839779

[B15] Frisoni G. B. Fox N. C. Jack C. R. Scheltens P. Thompson P. M. (2010). The clinical use of structural MRI in Alzheimer disease. *Nat. Rev. Neurol.* 6 67–77. 10.1038/nrneurol.2009.215 20139996 PMC2938772

[B16] Gasperini C. Rovaris M. Sormani M. P. Bastianello S. Pozzilli C. Comi G. (2001). Intra-observer, inter-observer and inter-scanner variations in brain MRI volume measurements in multiple sclerosis. *Mult. Scler. J.* 7 27–31. 10.1177/135245850100700106 11321190

[B17] Giavarina D. (2015). Understanding Bland Altman analysis. *Biochem. Medica* 25 141–151. 10.11613/BM.2015.015 26110027 PMC4470095

[B18] Good C. D. Johnsrude I. S. Ashburner J. Henson R. N. A. Friston K. J. Frackowiak R. S. J. (2001). A voxel-based morphometric study of ageing in 465 normal adult human brains. *Neuroimage* 14 21–36. 10.1006/nimg.2001.0786 11525331

[B19] Guo C. Niu K. Luo Y. Shi L. Wang Z. Zhao M. (2019). Intra-scanner and inter-scanner reproducibility of automatic white matter hyperintensities quantification. *Front. Neurosci.* 13:679. 10.3389/fnins.2019.00679 31354406 PMC6635556

[B20] Guptha S. H. Holroyd E. Campbell G. (2002). Progressive lateral ventricular enlargement as a clue to Alzheimer’s disease [6]. *Lancet* 359:2040. 10.1016/S0140-6736(02)08806-2 12076584

[B21] Halliday G. (2017). Pathology and hippocampal atrophy in Alzheimer’s disease. *Lancet Neurol.* 16 862–864. 10.1016/S1474-4422(17)30343-5 29029840

[B22] Harper L. Bouwman F. Burton E. J. Barkhof F. Scheltens P. O’Brien J. T. (2017). Patterns of atrophy in pathologically confirmed dementias: a voxelwise analysis. *J. Neurol. Neurosurg. Psychiatry* 88 908–916. 10.1136/jnnp-2016-314978 28473626 PMC5740544

[B23] Harper L. Fumagalli G. G. Barkhof F. Scheltens P. O’Brien J. T. Bouwman F. (2016). MRI visual rating scales in the diagnosis of dementia: evaluation in 184 post-mortem confirmed cases. *Brain* 139 1211–1225. 10.1093/brain/aww005 26936938 PMC4806219

[B24] Huppertz H.-J. Kröll-Seger J. Klöppel S. Ganz R. E. Kassubek J. (2010). Intra- and interscanner variability of automated voxel-based volumetry based on a 3D probabilistic atlas of human cerebral structures. *Neuroimage* 49 2216–2224. 10.1016/j.neuroimage.2009.10.066 19878722

[B25] Jack C. R. Albert M. S. Knopman D. S. McKhann G. M. Sperling R. A. Carrillo M. C. (2011). Introduction to the recommendations from the National Institute on Aging-Alzheimer’s Association workgroups on diagnostic guidelines for Alzheimer’s disease. *Alzheimers Dement.* 7 257–262. 10.1016/j.jalz.2011.03.004 21514247 PMC3096735

[B26] Jacobs H. I. L. Van Boxtel M. P. J. Heinecke A. Gronenschild E. H. B. M. Backes W. H. Ramakers I. H. G. B. (2012a). Functional integration of parietal lobe activity in early Alzheimer disease. *Neurology* 78 352–360. 10.1212/WNL.0b013e318245287d 22262753

[B27] Jacobs H. I. L. Van Boxtel M. P. J. Jolles J. Verhey F. R. J. Uylings H. B. M. (2012b). Parietal cortex matters in Alzheimer’s disease: an overview of structural, functional and metabolic findings. *Neurosci. Biobehav. Rev.* 36 297–309. 10.1016/j.neubiorev.2011.06.009 21741401

[B28] Jain S. Sima D. M. Ribbens A. Cambron M. Maertens A. Van Hecke W. (2015). Automatic segmentation and volumetry of multiple sclerosis brain lesions from MR images. *NeuroImage Clin.* 8, 367–375. 10.1016/j.nicl.2015.05.003 26106562 PMC4474324

[B29] Koo T. K. Li M. Y. (2016). A guideline of selecting and reporting intraclass correlation coefficients for reliability research. *J. Chiropr. Med.* 15 155–163. 10.1016/j.jcm.2016.02.012 27330520 PMC4913118

[B30] Lindberg O. Walterfang M. Looi J. C. L. Malykhin N. Ostberg P. Zandbelt B. (2012). Hippocampal shape analysis in Alzheimer’s disease and frontotemporal lobar degeneration subtypes. *J. Alzheimers. Dis.* 30 355–365. 10.3233/JAD-2012-112210 22414571 PMC4862006

[B31] Magnotta V. A. Friedman L. (2006). Measurement of signal-to-noise and contrast-to-noise in the fBIRN multicenter imaging study. *J. Digit. Imaging* 19 140–147. 10.1007/s10278-006-0264-x 16598643 PMC3045184

[B32] Martinez-Torteya A. Rivera-Davila M. Celaya-Padilla J. M. Tamez-Peña J. G. Rodríguez-Cantú F. E. (2019). “Measuring hippocampal neuroanatomical asymmetry to better diagnose Alzheimer’s disease,” in *Proceedings of the SPIE-Intl Soc Optical Eng*, San Diego, CA, 28.

[B33] Maruszak A. Thuret S. (2014). Why looking at the whole hippocampus is not enough-a critical role for anteroposterior axis, subfield and activation analyses to enhance predictive value of hippocampal changes for Alzheimer’s disease diagnosis. *Front. Cell. Neurosci.* 8:95. 10.3389/fncel.2014.00095 24744700 PMC3978283

[B34] McGraw K. O. Wong S. P. (1996). Forming inferences about some intraclass correlation coefficients. *Psychol. Methods* 1 30–46. 10.1037/1082-989X.1.1.30

[B35] McKhann G. M. Knopman D. S. Chertkow H. Hyman B. T. Jack C. R. Kawas C. H. (2011). The diagnosis of dementia due to Alzheimer’s disease: recommendations from the National Institute on Aging-Alzheimer’s Association workgroups on diagnostic guidelines for Alzheimer’s disease. *Alzheimers Dement.* 7 263–269. 10.1016/j.jalz.2011.03.005 21514250 PMC3312024

[B36] Niemantsverdriet E. Ribbens A. Bastin C. Benoit F. Bergmans B. Bier J. C. (2018). A retrospective Belgian Multi-Center MRI biomarker study in Alzheimer’s disease (REMEMBER). *J. Alzheimers Dis.* 63 1509–1522. 10.3233/JAD-171140 29782314 PMC6004934

[B37] Pemberton H. G. Goodkin O. Prados F. Das R. K. Vos S. B. Moggridge J. (2021). Automated quantitative MRI volumetry reports support diagnostic interpretation in dementia: a multi-rater, clinical accuracy study. *Eur. Radiol.* 31 5312–5323. 10.1007/s00330-020-07455-8 33452627 PMC8213665

[B38] Peng G. Wang J. Feng Z. Liu P. Zhang Y. He F. (2016). Clinical and neuroimaging differences between posterior cortical atrophy and typical amnestic Alzheimer’s disease patients at an early disease stage. *Sci. Rep.* 6:29372. 10.1038/srep29372 27377199 PMC4932506

[B39] Rathakrishnan B. G. Murali Doraiswamy P. Petrella J. R. (2014). Science to practice: translating automated brain MRI volumetry in Alzheimer’s disease from research to routine diagnostic use in the work-up of dementia. *Front. Neurol.* 4:216. 10.3389/fneur.2013.00216 24409168 PMC3885875

[B40] Revelle W. (2012). An Introduction to Psychometric Theory with Applications in R. *Personal. Proj.* 1–262. Available at: papers://dee23da0-e34b-4588-b624-f878b46d7b3d/Paper/p728.

[B41] Sarica A. Vasta R. Novellino F. Vaccaro M. G. Cerasa A. Quattrone A. (2018). MRI asymmetry index of hippocampal subfields increases through the continuum from the mild cognitive impairment to the alzheimer’s disease. *Front. Neurosci.* 12:576. 10.3389/fnins.2018.00576 30186103 PMC6111896

[B42] Sawyer R. P. Rodriguez-Porcel F. Hagen M. Shatz R. Espay A. J. (2017). Diagnosing the frontal variant of Alzheimer’s disease: a clinician’s yellow brick road. *J. Clin. Mov. Disord.* 4:2. 10.1186/s40734-017-0052-4 28265458 PMC5333400

[B43] Scahill R. I. Frost C. Jenkins R. Whitwell J. L. Rossor M. N. Fox N. C. (2003). A longitudinal study of brain volume changes in normal aging using serial registered magnetic resonance imaging. *Arch. Neurol.* 60 989–994. 10.1001/archneur.60.7.989 12873856

[B44] Schippling S. Ostwaldt A. C. Suppa P. Spies L. Manogaran P. Gocke C. (2017). Global and regional annual brain volume loss rates in physiological aging. *J. Neurol.* 264 520–528. 10.1007/s00415-016-8374-y 28054131

[B45] Shinohara R. T. Oh J. Nair G. Calabresi P. A. Davatzikos C. Doshi J. (2017). Volumetric analysis from a harmonized multisite brain MRI study of a single subject with multiple sclerosis. *AJNR. Am. J. Neuroradiol.* 38 1501–1509. 10.3174/ajnr.A5254 28642263 PMC5557658

[B46] Shrout P. E. Fleiss J. L. (1979). Intraclass correlations: uses in assessing rater reliability. *Psychol. Bull.* 86 420–428. 10.1037/0033-2909.86.2.420 18839484

[B47] Sima D. M. Horáková D. Nguyen A.-L. Van Hecke W. Kalincik T. Barnett M. H. (2019). Assessing the reliability of longitudinal MRI examinations in multiple sclerosis follow-up. *ECTRIMS Online Libr.* 278907:547.

[B48] Sluimer J. D. Vrenken H. Blankenstein M. A. Fox N. C. Scheltens P. Barkhof F. (2008). Whole-brain atrophy rate in Alzheimer disease: identifying fast progressors. *Neurology* 70 1836–1841. 10.1212/01.wnl.0000311446.61861.e3 18458218

[B49] Sperling R. A. Aisen P. S. Beckett L. A. Bennett D. A. Craft S. Fagan A. M. (2011). Toward defining the preclinical stages of Alzheimer’s disease: recommendations from the National Institute on Aging-Alzheimer’s Association workgroups on diagnostic guidelines for Alzheimer’s disease. *Alzheimers Dement.* 7 280–292. 10.1016/j.jalz.2011.03.003 21514248 PMC3220946

[B50] Stöckl D. Rodríguez Cabaleiro D. Van Uytfanghe K. Thienpont L. M. (2004). Interpreting method comparison studies by use of the Bland-Altman plot: reflecting the importance of sample size by incorporating confidence limits and predefined error limits in the graphic [3]. *Clin. Chem.* 50 2216–2218. 10.1373/clinchem.2004.036095 15502104

[B51] Struyfs H. Sima D. M. Wittens M. Ribbens A. Pedrosa de Barros N. Phan T. V. (2020). Automated MRI volumetry as a diagnostic tool for Alzheimer’s disease: validation of icobrain dm. *Neuroimage Clin.* 26:102243. 10.1016/j.nicl.2020.102243 32193172 PMC7082216

[B52] Studholme C. Hill D. L. G. Hawkes D. J. (1999). An overlap invariant entropy measure of 3D medical image alignment. *Pattern Recognit.* 32 71–86. 10.1016/S0031-3203(98)00091-0

[B53] Trefler A. Sadeghi N. Thomas A. G. Pierpaoli C. Baker C. I. Thomas C. (2016). Impact of time-of-day on brain morphometric measures derived from T1-weighted magnetic resonance imaging. *Neuroimage* 133 41–52. 10.1016/j.neuroimage.2016.02.034 26921714 PMC5602560

[B54] Trevethan R. (2017). Intraclass correlation coefficients: clearing the air, extending some cautions, and making some requests. *Heal. Serv. Outcomes Res. Methodol.* 17 127–143. 10.1007/s10742-016-0156-6

[B55] Vemuri P. Jack C. R. (2010). Role of structural MRI in Alzheimer’s disease. *Alzheimers Res. Ther.* 2:23. 10.1186/alzrt47 20807454 PMC2949589

[B56] Vernooij M. W. Pizzini F. B. Schmidt R. Smits M. Yousry T. A. Bargallo N. (2019). Dementia imaging in clinical practice: a European-wide survey of 193 centres and conclusions by the ESNR working group. *Neuroradiology* 61 633–642. 10.1007/s00234-019-02188-y 30852630 PMC6511357

[B57] Vinke E. J. de Groot M. Venkatraghavan V. Klein S. Niessen W. J. Ikram M. A. (2018). Trajectories of imaging markers in brain aging: the Rotterdam Study. *Neurobiol. Aging* 71 32–40. 10.1016/j.neurobiolaging.2018.07.001 30077040

[B58] Wittens M. M. J. Sima D. M. Houbrechts R. Ribbens A. Niemantsverdriet E. Fransen E. (2021). Diagnostic performance of automated MRI volumetry by icobrain dm for Alzheimer’s disease in a clinical setting: a REMEMBER study. *J. Alzheimers Dis.* 1–17. 10.3233/jad-210450 pre-press 34334402 PMC8543261

[B59] Wolak M. E. Fairbairn D. J. Paulsen Y. R. (2012). Guidelines for estimating repeatability. *Methods Ecol. Evol.* 3 129–137. 10.1111/J.2041-210X.2011.00125.X

