# Supplementary material for: Inter- and Intra-Scanner Variability of Automated Brain Volumetry on Three Magnetic Resonance Imaging Systems in Alzheimer’s Disease and Controls
Source: Front Aging Neurosci. 2021 Oct 7;13:746982. doi: 10.3389/fnagi.2021.746982 (PMC8530224; doi:10.3389/fnagi.2021.746982)
Supplement: Supplementary file 1 [file Table_1.DOCX]

# **SUPPLEMENTARY MATERIAL**

**Supplementary Table 1: Absolute CNR values per MRI system.** Data is reported as mean ± SD. **CNR** = contrast to noise ratio **WM** = white matter. **GM** = gray matter.

| SCANNER COMPARISON | ABSOLUTE CNR VALUE – T1w WM/GM CONTRAST (mean ± SD) |
| --- | --- |
| GE | 3.47 ± 0.26 |
| Ingenia | 3.62 ± 0.23 |
| Achieva | 3.78 ± 0.26 |

**Supplementary Table 2:** Mixed model analysis results for brain structures showing an overall significant difference in dice similarity coefficient (DSC) values.

| **BRAIN STRUCTURES** | **Estimate** | **Std. error** | **z-value** | **Adjusted p-value (Bonferroni)** |
| --- | --- | --- | --- | --- |
| **HIPPOCAMPUS** |  | | | |
| Achieva – GE | 0.023191 | 0.004474 | 5.184 | 6.52e-07 |
| Achieva – Ingenia | 0.001914 | 0.004474 | 0.428 | 0.904 |
| Ingenia – GE | -0.021276 | 0.004474 | -4.756 | 5.93e-06 |
| **LEFT HIPPOCAMPUS** |  |  |  |  |
| Achieva – GE | 0.025424 | 0.006117 | 4.157 | 9.69e-05 |
| Achieva – Ingenia | -0.001600 | 0.006117 | -0.262 | 1 |
| Ingenia – GE | -0.027024 | 0.006117 | -4.418 | 2.99e-05 |
| **RIGHT HIPPOCAMPUS** |  |  |  |  |
| Achieva – GE | 0.020890 | 0.005409 | 3.862 | 0.000337 |
| Achieva – Ingenia | 0.005552 | 0.005409 | 1.026 | 0.914042 |
| Ingenia – GE | -0.015338 | 0.005409 | -2.836 | 0.013725 |

**Supplementary Table 3: Bland-Altman plot statistics.** Data presented as mean bias and 95% confidence interval (**Mean bias [95% CI]**), the upper limit of agreement and respective confidence intervals (**ULOA [95% CI]**) and the lower limit of agreement and respective confidence intervals (**LLOA [95% CI]**).

| **BRAIN STRUCTURES** | **Mean bias [95% CI]** | **ULOA [95% CI]** | **LLOA [95%CI]** |
| --- | --- | --- | --- |
| **WHOLE BRAIN** | -0.077 [-1.933 1.777] | 9.659 [6.453, 12.865] | -9.814 [-13.020, -6.609] |
| **GRAY MATTER** | 0.595 [-1.249, 2.440] | 10.277 [7.089, 13.465] | -9.086 [-12.274, -5.899] |
| **CORTICAL GRAY MATTER** | 0.807 [-1.007, 2.621] | 10.329 [7.194, 13.464] | - 8.714 [-11.849, -5.579] |
| **WHITE MATTER** | -0.673 [-2.903, 1.557] | 11.030 [7.176, 14.883] | -12.376 [-16.229, -8.523] |
| **FRONTAL CORTEX** | -0.127 [-0.943, 0.689] | 4.156 [2.746, 5.566] | -4.410 [-5.821, -3.000] |
| **PARIETAL CORTEX** | 0.157 [-1.008, 1.321] | 6.267 [4.255, 8.279] | -5.953 [-7.966, -3.942] |
| **TEMPORAL CORTEX** | 0.338 [-0.830, 1.505] | 6.466 [4.448, 8.484] | -5.790 [-7.808, -3.773] |
| **HIPPOCAMPUS, TOTAL** | 0.016 [-0.041, 0.073] | 0.316 [0.218, 0.415] | -0.284 [-0.382, -0.185] |
| **HIPPOCAMPUS, LEFT** | 0.010 [-0.046, 0.065] | 0.303 [0.206, 0.400] | -0.283 [-0.379, -0.186] |
| **HIPPOCAMPUS, RIGHT** | 0.006 [-0.021, 0.033] | 0.149 [0.102, 0.196] | -0.136 [-0.183, -0.089] |
| **LATERAL VENTRICLES** | 0.142 [-0.097, 0.393] | 1.433 [1.010, 1.856] | -1.137 [-1.156, -0.714] |

**Supplementary Table 4:** Mixed model analysis results for brain structures showing an overall significant difference in actual volumes

| **BRAIN STRUCTURES** | **Estimate** | **Std. error** | **z-value** | **Adjusted p-value (Bonferroni)** |
| --- | --- | --- | --- | --- |
| **WHOLE BRAIN** |  | | | |
| Achieva – GE | 2.432 | 1.563 | 1.557 | 0.359 |
| Achieva – Ingenia | -7.124 | 1.563 | -4.560 | 1.54e-05 |
| Ingenia – GE | -9.557 | 1.563 | -6.116 | 2.87e-09 |
| **GRAY MATTER** |  |  |  |  |
| Achieva – GE | -33.913 | 2.391 | -14.182 | <2e-16 |
| Achieva – Ingenia | -3.888 | 2.391 | -1.626 | 0.312 |
| Ingenia – GE | 30.025 | 2.391 | 12.556 | <2e-16 |
| **CORTICAL GRAY MATTER** |  |  |  |  |
| Achieva – GE | -30.243 | 2.120 | -14.267 | <2e-16 |
| Achieva – Ingenia | -4.084 | 2.120 | -1.927 | 0.162 |
| Ingenia – GE | -4.084 | 2.120 | 12.341 | <2e-16 |
| **WHITE MATTER** |  |  |  |  |
| Achieva – GE | 36.346 | 2.584 | 14.063 | <2e-16 |
| Achieva – Ingenia | -3.236 | 2.584 | -1.252 | 0.631 |
| Ingenia – GE | -39.582 | 2.584 | -15.315 | <2e-16 |
| **FRONTAL CORTEX** |  |  |  |  |
| Achieva – GE | -11.3497 | 0.6875 | -16.508 | <2e-16 |
| Achieva – Ingenia | -0.9303 | 0.6875 | -1.353 | 0.528 |
| Ingenia – GE | 10.4194 | 0.6875 | 15.155 | <2e-16 |
| **PARIETAL CORTEX** |  |  |  |  |
| Achieva – GE | -6.73856 | 0.77007 | -8.751 | <2e-16 |
| Achieva – Ingenia | -0.06111 | 0.77007 | -0.079 | 1 |
| Ingenia – GE | 6.67745 | 0.77007 | -0.079 | <2e-16 |
| **TEMPORAL CORTEX** |  |  |  |  |
| Achieva – GE | -2.7867 | 0.7407 | -3.762 | 0.000506 |
| Achieva – Ingenia | -1.7289 | 0.7407 | -2.334 | 0.058776 |
| Ingenia – GE | 1.0578 | 0.7407 | 1.428 | 0.459882 |
| **RIGHT HIPPOCAMPUS** |  |  |  |  |
| Achieva – GE | -0.083741 | 0.023876 | -3.507 | 0.001358 |
| Achieva – Ingenia | 0.007154 | 0.023876 | 0.300 | 1.000000 |
| Ingenia – GE | 0.090895 | 0.023876 | 3.807 | 0.000422 |
| **LATERAL VENTRICLLES** |  |  |  |  |
| Achieva – GE | -0.1177 | 0.2081 | -0.566 | 1.000000 |
| Achieva – Ingenia | 0.7752 | 0.2081 | 3.726 | 0.000585 |
| Ingenia – GE | 0.8929 | 0.2081 | 4.291 | 5.33e-05 |
